# Leveraging fourier-transform infrared spectroscopy for community drug checking to bridge clinical care and harm reduction services: case report

**DOI:** 10.1186/s13722-026-00697-9

**Published:** 2026-07-25

**Authors:** Elizabeth Znamierowski, Katherine Hill, Jenna L. Butner, Anna Stouffer, Jenna McDermit, Kenneth L. Morford

**Affiliations:** 1 Department of General Internal Medicine, Yale School of Medicine, New Haven, CT USA; 2 Department of Epidemiology of Microbial Diseases, Yale School of Public Health, New Haven, CT USA; 3 Internal Medicine, Yale New Haven Hospital, New Haven, CT USA

**Keywords:** Fourier-transform infrared spectroscopy, Fentanyl, Drug checking, Opioid use disorder, Case report

## Abstract

**Background:**

The opioid overdose crisis parallels the rise of numerous adulterants in the unregulated drug supply. Harm reduction programs have responded by expanding drug checking services, which chemically analyze street-based drugs. Using a clinical report, we demonstrate the utility of Fourier-transform infrared spectroscopy (FTIR) for engaging people who use drugs in simultaneous, tailored harm reduction and clinical services based on their drug-checking results.

**Case presentation:**

A woman with a history of fentanyl use (primarily fentanyl via inhalation) was found to be tachycardic during a follow-up visit at an addiction recovery clinic. The patient’s provider suggested she utilize local FTIR drug checking, which revealed that her substance contained a large amount of caffeine, and several other active components. Over the next month, the patient submitted six suspected fentanyl drug samples for testing. The patient shared the information with her partner and ultimately requested different drugs from her supplier. Additionally, FTIR results were communicated with the clinical team at the addiction recovery clinic and incorporated into ongoing discussions around the patient’s treatment plan.

**Conclusions:**

This case study suggests that low-barrier FTIR drug analysis serves as a valuable tool at the intersection of harm reduction and clinical care. Point-of-care drug checking allows clinicians to engage patients in discussion around etiology of wounds, complications of polysubstance use, and more. By equipping individuals who use drugs with real-time information regarding their substance(s), FTIR restores individual decision-making agency and autonomy. Additionally, drug checking fosters peer knowledge sharing-potentially leading to upstream changes to the unregulated drug supply.

## Background

### The opioid overdose crisis

The United States (US) overdose crisis is increasingly recognized in four distinct waves, with high potency synthetic opioids like fentanyl and its analogues attributed to rising overdose rates beginning in the 2010s [1]. The shift from prescription opioid deaths (wave one), to polysubstance related deaths (wave four) has reflected an increasingly unpredictable synthetic drug supply [1]. Attempts at regulation of the opioid supply, have driven US markets to shift to unregulated, inexpensive and increasingly potent alternatives [2,3]. Unregulated fentanyl is often mixed or “cut” with other substances to “bulk” product, enhance/prolong the fentanyl effects, or dilute potent fentanyl/analogues among other reasons. [4] For people who use drugs (PWUD), adulterants are frequently unknown to consumers, and can alter drug effects and dramatically increase health risks [5–7]. For instance, in Connecticut (CT), fentanyl was involved in 758 of 989 (76.6%) overdose deaths in 2024, with xylazine (a veterinary sedative and common adulterant of concern) detected in nearly half of postmortem toxicology reports [7,8]. Even seemingly benign additives (i.e., baking soda, caffeine) may cause harm, especially to those with underlying health conditions [9].

### Community-based drug checking

Drug checking offers a harm reduction approach to address the uncertain composition of street drugs by identifying the chemical composition of unregulated substances [10]. Harm reduction programs commonly use low-cost test strips that detect specific substances (e.g., xylazine), while more advanced technologies, such as Fourier-transform infrared spectroscopy (FTIR), provide a more in-depth analysis of a substance’s composition [11]. Although urine test strips are well studied in clinical settings, the utility of FTIR as a community-based point-of-care clinical tool remains underexplored. The utilization of FTIR as a point-of-care clinical tool has potential to be important for building a differential diagnosis and contextualizing patient specific substance use in a time when the drug supply changes rapidly.

## Caffeine in the unregulated supply

Caffeine is a common bulking agent in the unregulated opioid supply, potentially added to bulk product, enhance the effects of the opioid component or facilitate vaporization of the drug [12,13]. Given the fourth wave of the opioid crisis, and increasing interest in polysubstance use (specifically that of stimulants and opioids), it is possible that caffeine may mimic desired stimulant effects [14]. While moderate caffeine intake is generally safe, it can increase heart rate and blood pressure, particularly when combined with other stimulants or in individuals with underlying cardiovascular conditions [15–19]. Preliminary data from FTIR-based drug checking in New Haven, CT in 2025 found caffeine in 21% of opioid samples submitted as “fentanyl/heroin/dope”, [20] underscoring the need for real-time information and ongoing conversations with individuals around the unpredictably of the drug supply.

## Case presentation

A 63-year-old cisgender female with a history of opioid use disorder on long-acting injectable buprenorphine (Sublocade, 300 mg monthly), and intermittent fentanyl use via inhalation (~ 3 bags/day) presented to her addiction recovery clinic for follow-up. She reported hot flashes, palpitations, and the feeling of “crawling out of my skin.” Her last use of fentanyl was one bag, earlier that morning. Upon exam, she was tachycardic (110–150 bpm) and hypertensive (153/93 mmHg) (see Table 1). Of note, a standard urine drug screen (UDS) was ordered, which did not include fentanyl as a separate test. The patient had previously positive fentanyl-positive UDS results from December of 2024 and again in May of 2025.

**Table 1:** Relevant clinical findings

Medical History		
Opioid use disorder (OUD) on long-acting injectable buprenorphine (Sublocade, 300 mg monthly), chronic benzodiazepine use (undergoing a medically managed gradual clonazepam taper), tobacco use disorder (in remission), alcohol use, post-traumatic stress disorder, major depressive disorder, asthma, chronic obstructive pulmonary disease, hepatitis B (resolved), urinary retention, and constipation.		
**Physical Examination Findings 3/11/2025**		
Mildly anxious appearance Easily engageable Mildly diaphoretic Normal work of breathing Normal ambulation		
**Vital Signs 3/11/2025**		
Blood pressure 153/93 Heart rate 110–150 s		
**Tests & Lab Results**		
Urine drug screen 3/11/2025* ** Positives**: aminoclonazepam (498), benzodiazepines, buprenorphine (1,197), norbuprenorphine (2000) ** Negatives**: alphahydroxyalprozalam, alphahydroxymidazolam, alphahydroxytriazolam, amphetamines, cocaine, heroin, hydroxyethylflurazepam, lorazepam, [cannabis], methadone, nordiazepam, opiates,oxazepam, oxycodone, temazepam, barbiturates, phencyclidine **EKG 3/11/2025** sinus tachycardia and right bundle branch block (RBB present since 2019)		

*Of note, on 3/11/25 only a standard UDS was ordered, which did not include fentanyl as a separate test

Her past medical history included chronic benzodiazepine use (prolonged taper), alcohol use, post-traumatic stress disorder, depression, chronic obstructive pulmonary disease, and resolved hepatitis B. She lived in stable housing with her husband, who was on methadone maintenance therapy and used unregulated fentanyl. She had not previously engaged with any harm reduction services.

**Fig. 1:**
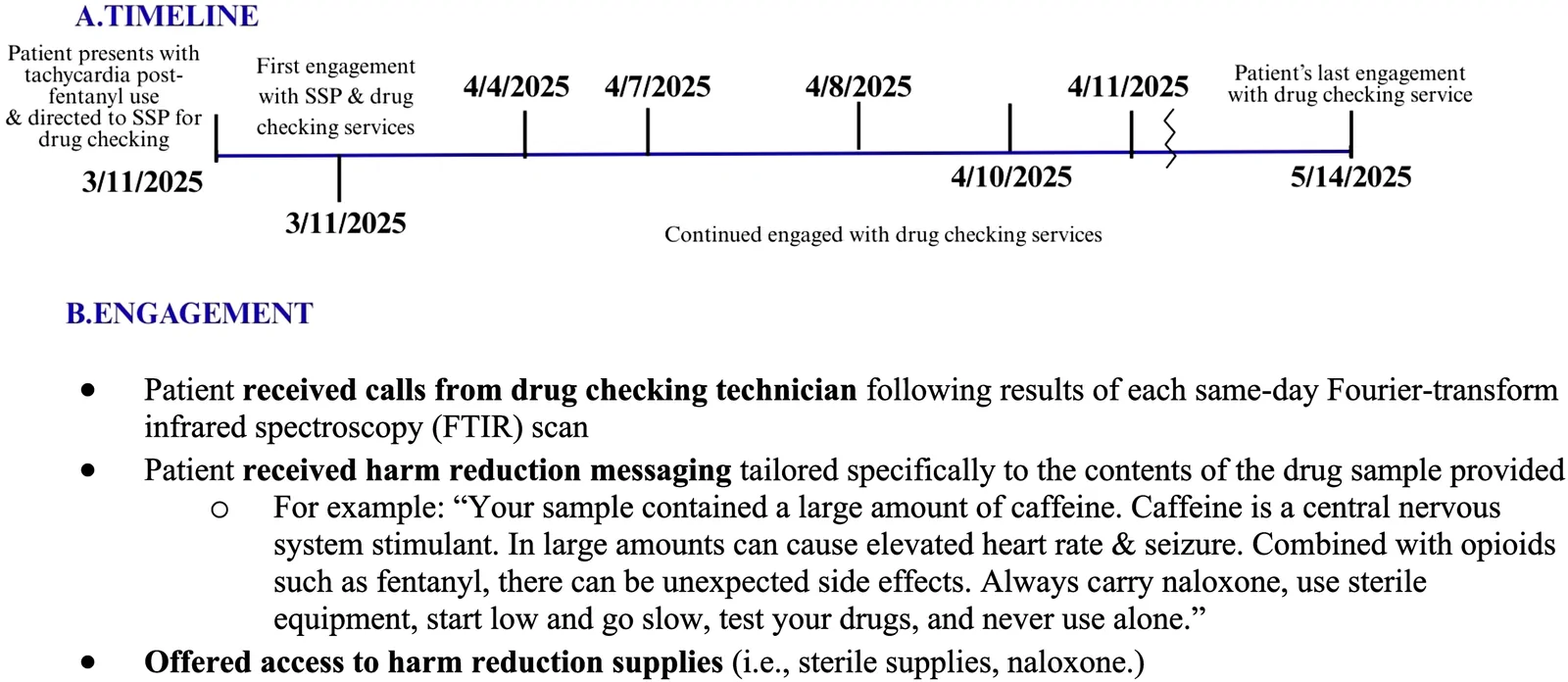
Timeline and description of patient engagement with addiction treatment team and drug checking services

Following a referral by her addiction medicine provider, the patient presented to the local syringe services program (SSP) for drug checking. FTIR analysis of a sample from the same batch she had used that morning revealed caffeine as the major component, with trace fentanyl and other excipients (see Fig. 1). The patient observed the testing process, discussing the benefits and limitations of FTIR [21], as well as substance-specific harm reduction strategies (e.g., start with a small amount, avoid using alone). Given the presence of caffeine, the FTIR technician/Advanced Practice Nurse Practitioner discussed that the stimulant effects of caffeine in the patient’s sample may have contributed to her tachycardia. Results were shared with the patient’s addiction treatment team to inform ongoing care and provided additional differentials for plausible contributory factors of the patient’s tachycardia. The clinical team discussed several potential contributors to the patient’s tachycardia, including possible opioid withdrawal (though less likely given her monthly Sublocade dosing), unintentional stimulant exposure from the drug supply, hypovolemia and possible albuterol-related effects.

The patient returned to the SSP for additional testing of five subsequent samples. Figure 1, **panel A** provides a timeline of the case presentation and subsequent drug checking interactions.

The patient expressed a desire to track her supply over time and to obtain less contaminated fentanyl products. Figure 1 provides engagement details (**panel B**), and longitudinal drug checking results (Table 2**)** of the submitted samples provided by the patient. Additionally, the patient received follow up care at her addiction recovery clinic, with continued Sublocade, and ongoing counseling and support. At follow-up visits, she reported increased motivation to reduce/stop substance use after engaging with drug checking services and learning her supply was adulterated. Notably, her tachycardia had resolved by these visits. The drug checking results may have been useful not only in diagnostic reasoning (i.e. Caffeine exposure as a contributor), but also subsequent motivational interviewing and harm reduction counseling.

**Table 2:** Drug checking results

Date	Purchased As	Results
3/11/2025	Fentanyl	**FTIR**: Caffeine (major), Mannitol (minor), Fentanyl (minor)
4/4/2025	Fentanyl	**FTIR**: Caffeine (major), Procaine (major), BTMPS* (minor), Fentanyl (trace)
4/7/2025	Fentanyl	**FTIR**: Mannitol (major), Caffeine (major), Fentanyl (minor), BTMPS* (minor/trace)
4/8/2025	Fentanyl	**FTIR**: Mannitol (major), Fentanyl (minor), BTMPS* (minor)
4/10/2025	Fentanyl	**FTIR**: Caffeine (major), Mannitol (major), Fentanyl (minor), BTMPS* (unknown)
4/11/2025	Fentanyl	**FTIR**: Mannitol (major), Caffeine (major), Fentanyl (minor), BTMPS* (minor/trace)
4/11/2025	Fentanyl	**FTIR**: Procaine (major), Mannitol (major), Fentanyl (minor), Caffeine (minor)
5/14/2025	Fentanyl	**GCMS**: Caffeine 177, Tetramethyl-4-AP (TM-4-AP) 100, Tetramethylnorfentanyl (TMNF) 33, Xylazine 24, Fentanyl 10, 4-ANPP 2 **

*bis(2,2,6,6-tetramethyl-4-piperidyl)sebacate**Secondary results courtesy of CT Public Health Laboratory Gas Chromatography-Mass Spectrometry (GCMS) allow determination of the relative concentration of active substances in submitted samples

Reflecting on her initial referral to the SSP drug checking program, the patient stated, *“That one time with the caffeine… [the provider] told me to go over there and have it checked. As long as people feel safe coming to you—but a lot don’t*,* because what if the cops find out? They don’t know you won’t call the cops.”* Her experience highlights the importance of confidentiality and trust in engagement with harm reduction services. Over time, this relationship led her to bring her husband to the SSP to observe the drug checking process himself. Additionally, after seeing the fluctuations in her product, she also wanted her supplier of drugs to engage with the service. She stated, *“I told him [i.e.*,* my drug supplier]*,* ‘go there [i.e.*,* the SSP] and just check out what you have’ because he gave me some bad shit one time when I first met him*,* and it knocked me on my ass…it knocked me out cold*,* no one could get a hold of me.”* This patient’s desire to share drug checking knowledge with others and engage them in drug checking services reflects the downstream community benefits possible from such an intervention. Existing literature supports the protective and positive impacts of shared community knowledge surrounding drug checking [22,23].

## Discussion

This case underscores the value of FTIR, not simply as a diagnostic tool, [24] but also as a means of opening clinical dialogue, fostering care engagement, mitigating overdose and other deleterious effects of use, and restoring agency to people who use drugs (PWUD). In this instance, FTIR drug checking provided a potential tangible explanation for a patient’s unexpected physiological symptoms– namely, tachycardia possibly caused by caffeine-adulterated fentanyl – and served as an entry point into harm reduction services for a person with no prior engagement. In the setting of this patient’s complex clinical history, other plausible contributory factors for tachycardia could include withdrawal from her prolonged benzodiazepine taper, and opioid withdrawal (although less likely given her same-day Sublocade dosing), underscoring the importance of having a panel of available tests to provide a broader differential diagnosis for symptomology. Additionally, based the prevalence of caffeine in the New Haven opioid supply, there is a significant possibility that the patient had been exposed to caffeine previously. The patient’s tachycardia may have been associated with exposure to a batch containing a particularly high concentration of caffeine. Clinically, the dose/amount of caffeine exposure rather than presence alone may be significant. While we do not have secondary Gas Chromatography-Mass Spectrometry (GCMS) results for the 3/11/25 fentanyl sample, we can utilize FTIR concentration estimates for component concentration. FTIR component designations are generally major (30–100%), minor (10–29%), or trace (< 10 − 5%). FTIR concentrations are less precise than GCMS and are technician dependent but can indicate an estimated percent composition. In this case, the patient’s 3/11/25 sample contained a major component designation for caffeine, which may indicate that she experienced exaggerated symptoms given the amount of caffeine in the sample.

While FTIR has limitations, including a higher limit of detection than laboratory-based analyses [21] and significant up-front financial costs [11], it offers unique opportunities to engage PWUD when embedded in community-based harm reduction settings where healthcare and outreach are closely integrated. FTIR can provide insight into the unregulated drug supply and provide both PWUD and providers with information otherwise inaccessible via current clinical testing. In cases where FTIR may not be able to reliably provide explanations for physiological symptoms (i.e. cases involving novel adulterants, drug-drug interactions, or substances under the limit of detection), there remains great utility in engaging PWUD as agents in their own health.

In this case, follow-up phone calls with a patient accessing community-based drug checking services served not only to relay information about the chemical composition of a submitted sample but also to create touchpoints for personalized harm reduction messaging tailored to the specific drugs, adulterants, and excipients identified. Provision of non-stigmatizing, person-centered services positions PWUD as experts in their own care, encourages rapport and trust, and delivers timely information on the substances they use. Collaboration with clinical teams is particularly valuable for contextualizing drug-checking results alongside medical history. In this case, current evidence does not suggest that moderate caffeine intake causes significant cardiac effects, but it can produce unwanted physical symptoms. In an unregulated and volatile drug supply, it is imperative that clinicians listen to patients if they express unwanted side effects of their substance use. Furthermore, the presence of caffeine in a sample may have important implications for individuals using other substances [15,16]. Integrating clinical insight into harm reduction conversations allows for individualized risk assessment, helping patients make better-informed decisions about their substance use.

Beyond its informative function, community-based drug checking re-centers agency into the hands of PWUD by recognizing them as experts in their own experiences and as essential contributors to public health knowledge. Through confidential, bidirectional communication and access to real-time, actionable data, individuals retain autonomy and are empowered to make informed decisions as consumers navigating an unpredictable, dangerous, and unregulated drug market. Drug checking transforms PWUD from passive recipients of services into active participants in both their own care and broader community awareness and safety. This case illustrates a patient’s demonstrated trust and ongoing engagement, eventually leading her to share local drug supply trends with her social networks, contributing to collective knowledge and safety.

Despite the growing use of drug checking tools like fentanyl test strips, FTIR, and laboratory secondary testing (e.g., GCMS and Liquid Chromatography-Quadrupole Time-of-Flight), there remains a notable gap in the literature regarding the use of point-of-care drug checking in clinical contexts to support differential diagnoses. Future research should explore how FTIR-based community drug checking can be leveraged not only for individual-level harm reduction, including bolstering of autonomy and empowerment, but also for enhancing healthcare and patient-centered approaches to mitigating drug-related harms.

## Informed consent

Consent was received from the patient using Yale New Haven Health’s Authorization for Medical Case Study and Publication form. The patient was involved from the conception of the case study through final drafting and submission. Direct quotations utilized in the case presentation were collected after routine visits for drug checking. Quotations were transcribed with permission of the patient, solely for the purposes of this case report and were not recorded.

## Data Availability

No datasets were generated or analysed during the current study.

## Abbreviations

CT: Connecticut
FTIR: Fourier-transform infrared spectroscopy
GCMS: Gas Chromatography-Mass Spectrometry
PWUD: People who use drugs
SSP: Syringe services program
UDS: Urine drug screen
